# Attachment and Children’s Biased Attentional Processing: Evidence for the Exclusion of Attachment-Related Information

**DOI:** 10.1371/journal.pone.0103476

**Published:** 2014-07-25

**Authors:** Eva Vandevivere, Caroline Braet, Guy Bosmans, Sven C. Mueller, Rudi De Raedt

**Affiliations:** 1 Department of Developmental, Personality and Social Psychology, Ghent University, Gent, Belgium; 2 Parenting and Special Education Research Unit, Leuven, Belgium; 3 Department of Experimental Clinical and Health Psychology, Ghent University, Ghent, Belgium; Erasmus University Rotterdam, Netherlands

## Abstract

Research in both infants and adults demonstrated that attachment expectations are associated with the attentional processing of attachment-related information. However, this research suffered from methodological issues and has not been validated across ages. Employing a more ecologically valid paradigm to measure attentional processes by virtue of eye tracking, the current study tested the defensive exclusion hypothesis in late childhood. According to this hypothesis, insecurely attached children are assumed to defensively exclude attachment-related information. We hypothesized that securely attached children process attachment- related neutral and emotional information in a more open manner compared to insecurely attached children. Sixty-two children (59.7% girls, 8–12 years) completed two different tasks, while eye movements were recorded: task one presented an array of neutral faces including mother and unfamiliar women and task two presented the same with happy and angry faces. Results indicated that more securely attached children looked longer at mother’s face regardless of the emotional expression. Also, they tend to have more maintained attention to mother’s neutral face. Furthermore, more attachment avoidance was related to a reduced total viewing time of mother’s neutral, happy, and angry face. Attachment anxiety was not consistently related to the processing of mother’s face. Findings support the theoretical assumption that securely attached children have an open manner of processing all attachment-related information.

## Introduction

Infants are biologically predisposed to form attachment bonds to an available caregiver [2]. Already in childhood, specific attachment-related individual differences appear to translate in specific attachment-related expectations [3]. This internalization process is seen as a crucial mechanism underlying the early caregiver-child relationship [4]. However, it is still not clear how expectations about care and comfort operate in childhood. Bowlby [5] already pointed out that specifically the processing of social information is affected by attachment expectations. To date, empirical research confirmed that differences in attachment influence the processing of social information [6]. This mechanism was first studied in infancy [3], later in adults [7] and to a lesser extent in childhood [8]. The current study aims to expand the existing knowledge on how different attachment expectations guide information processing in late childhood using a new paradigm.

### The internal working model and information processing

A core assumption of attachment theory is that repeated experiences with attachment figures are stored into mental representations or internal working models (IWM). This internalizing process takes off early in life and provides the individual scripts which form the way people think, feel, and behave in close relationships [2]. To date, researchers tried to map the content of internal working models and robustly revealed individual differences. Three different attachment categories were inferred in infants from observations during the Strange Situation procedure, 1) secure attachment, 2) insecure-ambivalent attachment, and 3) insecure-avoidant attachment [9]. Later on, interviews and self-reports were developed to map attachment styles in older age groups, leading to comparable categorical but more recently also dimensional models. Statistical evidence has supported that dimensional models are valid models to map attachment differences [10]. One dimensional model, developed by Brennan, Clark, & Shaver [11], assumes people to vary on an anxiety and avoidance dimension. Attachment anxiety refers to the degree to which a person worries that their attachment figure will not be available or adequately responsive in times of need. Attachment avoidance reflects the extent to which an individual distrusts his attachment figure’s availability and strives to maintain autonomy and emotional distance from them [12]. These individual differences in attachment style not only reflect internalized expectations about the caregiver, but also affect the way in which information is processed [6].

Attachment theorists have formulated specific predictions on how attachment expectations might already influence the earliest stages of information processing, namely the attentional processing of attachment-related information [13]. The defensive exclusion hypothesis [1] suggests that insecurely attached individuals will filter out all information related to his or her attachment figure, as this is associated with psychological pain. Even positive attachment-related information is seen as painful, as it would trigger the notion that they only had a few or no positive experiences with their caregiver. This selective processing, defined as a bias, is protecting the insecurely attached individual from re-experiencing distress experienced in the past and limits the activation of the attachment system [1]. In contrast, securely attached persons are hypothesized to openly process all attachment-related information, including negative attachment-related information. This negative information is less distressing for securely attached individuals than for insecurely attached individuals. When receiving negative attachment-related information, securely attached individuals hold the belief that the attachment figure is safe and still trust in the long-term availability of their attachment figure. Moreover, when they do feel upset when exposed to negative attachment-related information, securely attached individuals have developed, through former interactions with their attachment figure, adaptive regulation skills to independently cope with the distress [14]. Although this seems a very important theoretical statement, it has not extensively been researched in childhood. Therefore, in the present study we will measure whether neutral, negative, and positive attachment-related information is indeed suppressed by insecurely attached children.

### Eye movements in the study of attentional biases

In the current study we will examine attentional biases in the processing of attachment-relevant information for neutral, negative, and positive stimuli, measured by a naturalistic paradigm. The relations between attachment and attentional biases have mostly been observed in adults using cognitive reaction-time tasks, such as modified Stroop [15], probe-detection [16], or exogenous cueing paradigms [7]. These studies confirm that more insecurely attached adults defensively exclude psychological painful attachment-related information. Attentional biases in childhood are studied less extensively and research in childhood using experimental reaction-time paradigms does not confirm the defensive exclusion hypothesis [8]. Bosmans and colleagues [8] found evidence for an attentional bias towards mother in the low securely attached group. No effect was found for more securely attached children. However, reaction times are confounded by other non-attention processes such as motor activity and response selection [17]. Furthermore, these paradigms are measuring attention at a very specific moment, immediately after the presentation of the emotional stimulus, but are not suitable for capturing the course of attention over longer time periods [18]. Tracking eye movements could overcome these concerns and can be seen as a proxy of attention since they are functionally related to each other and share the same functional anatomical areas in the human brain [19]. The eye fixations correspond to the information being internally processed and the duration of the fixation is related to the time needed to encode and process information [20]. Shifts in fixation positions closely follow and are guided by shifts in attentional focus [21]. Furthermore, eye tracking methodology is ideally suited for assessing continuously the visual gaze for a longer period.

Few studies have used eye-tracking to investigate the impact of internal working models of attachment on the information processing of attachment-related information. Main et al. (1985) showed six-year-old children a picture of a family and two observers rated whether they had an open manner of processing the picture (e.g. accepted the picture, smiled, and showed interest) or whether they avoided the picture or expressed unhappiness. Secure attachment (at 12 months measured by the strange situation procedure) was related to a more open processing of attachment-related information. Furthermore, insecure-avoidant children avoided the photographs more. Further, to our knowledge, only one study additionally focused on the processing of positive and negative attachment-related information. Kirch and Cassidy [22] varied the valence of attachment-related pictures given to the child. Children (45 months) were shown six sets of three mother-child drawings; each set consisted of a positive, negative, and neutral interaction. Eye movements were videotaped through a one-way mirror and an observer coded the direction of eye movements and the fixation time. Avoidantly attached children looked away from all attachment-related drawings more than secure and insecure-ambivalent children. No differences were found depending on the valence of the interactions. During a second task, children were given both an attachment-relevant positive interaction and a non-attachment-relevant neutral interaction. Compared to secure children, both insecure-avoidant, and -ambivalent children looked away more often from the positive attachment-relevant drawings. Hence, both studies have revealed that eye movement registration can be used to study how different attachment expectancies modulate the attentional processing of attachment-related information.

### The present study

The present study expands eye-tracking research in attachment by using continuous eye tracking to unobtrusively record the exact position of the person’s eye movements. Using the validated paradigm of Eizenman and colleagues [17], children will be shown multiple photographs with faces of their mother and unfamiliar women for a relatively long period (task one 10 sec./task two 8 sec.). These photographs will compete simultaneously for the child’s attention, enabling us to measure total viewing time, maintained attention, and relative visit frequency. Using faces with an idiosyncratic meaning, instead of general faces, increases the interpersonal relevance [7] and ecological validity of the study.

In task one, neutral facial expressions of mother and unfamiliar women will be used. Based on the defensive exclusion theory, we hypothesize that both anxiously and avoidantly attached children will be characterized by less total viewing time, less maintained attention and less visits on mother’s neutral face (relative to faces of unfamiliar women). At the same time, securely attached children are hypothesized to be characterized by an open processing of attachment-relevant information, leading to a longer total viewing time, more maintained attention, and more visits on mother’s neutral faces (relative to faces of unfamiliar women).

In the second task, emotional stimuli representing both negative and positive attachment-related information will be introduced to empirically substantiate the assumption that both positive and negative information will be filtered out of attention by insecurely attached children. In contrast to the study of Kirch and Cassidy [22], which used attachment scenes, emotional faces were shown as these stimuli could signal the emotional state of others more prominently than emotional scenes. Facial expressions are one of the most important types of emotional information encountered in social interaction [23] and are likely activators of the attachment system. Anger and happiness are relevant to study since both signal an invitation for social interaction. Happy emotional expressions invite the perceiver to approach the person, while anger demands conflict resolution [24]. It is hypothesized that more insecurely attached children will exclude, defensively, both positive and negative expressions by paying less attention to these maternal faces. More securely attached children on the other hand are hypothesized to openly process these emotional faces of mother, since both positive and negative emotional expressions of mother inform the child about mother her inner state and their relation. For more securely attached children, we hypothesize longer viewing times, more maintained attention, and more visits on mother’s happy and angry face (relative to the faces of unfamiliar women). Hence, the same information processing biases were expected for neutral, negative, and positive emotional expressions of mother.

## Methods

### Participants

A total of 68 children (58.28% girls), ages ranged from 8 to 12 years (*M* = 10.25, *SD* = .96), were recruited from elementary schools. Six children have been excluded due to insufficient gaze recording (<50%) (*N* = 4) and due to errors in the study-slides (*N* = 2). A total of 62 children (59.7% girls), ages ranged from 8 to 12 years (*M* = 10.72, *SD* = .96), are included in the study. Regarding parental level, 50% of the mothers had a master’s degree, 37.1% a bachelor degree, 11.3% a high school degree, and 1.6% had no school degree. Forty-seven participants (75.8%) were from intact families, whereas 22.6% of the participants were from divorced families and one child (1.6%) had a deceased parent. According to the mother, 69.4% of children had mainly contact with her during the first year of life, whereas 30.6% had equally contact with both parents during the first year of life.

### Ethics Statement

The study was approved by the Institutional Board of the Faculty of Psychology at Ghent University. Participating parents gave their informed consent for both taking photos and the test procedure for their child. Photos of participants, by which informed consent for usage was provided, were used in the study slides.

### Materials and apparatus

#### Attentional tasks

**Figure 1:**
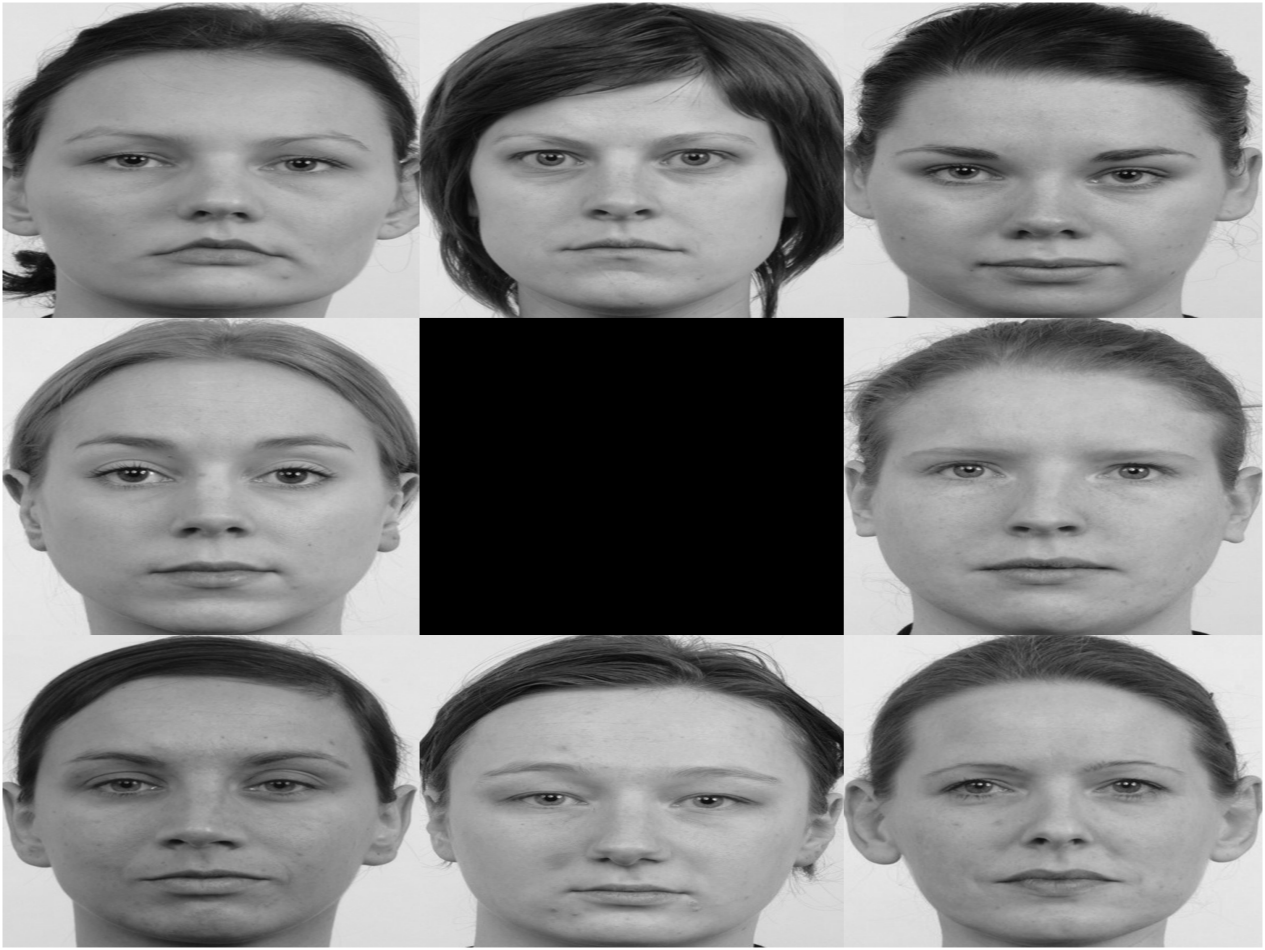
Example of a study slide task one.

**Figure 2:**
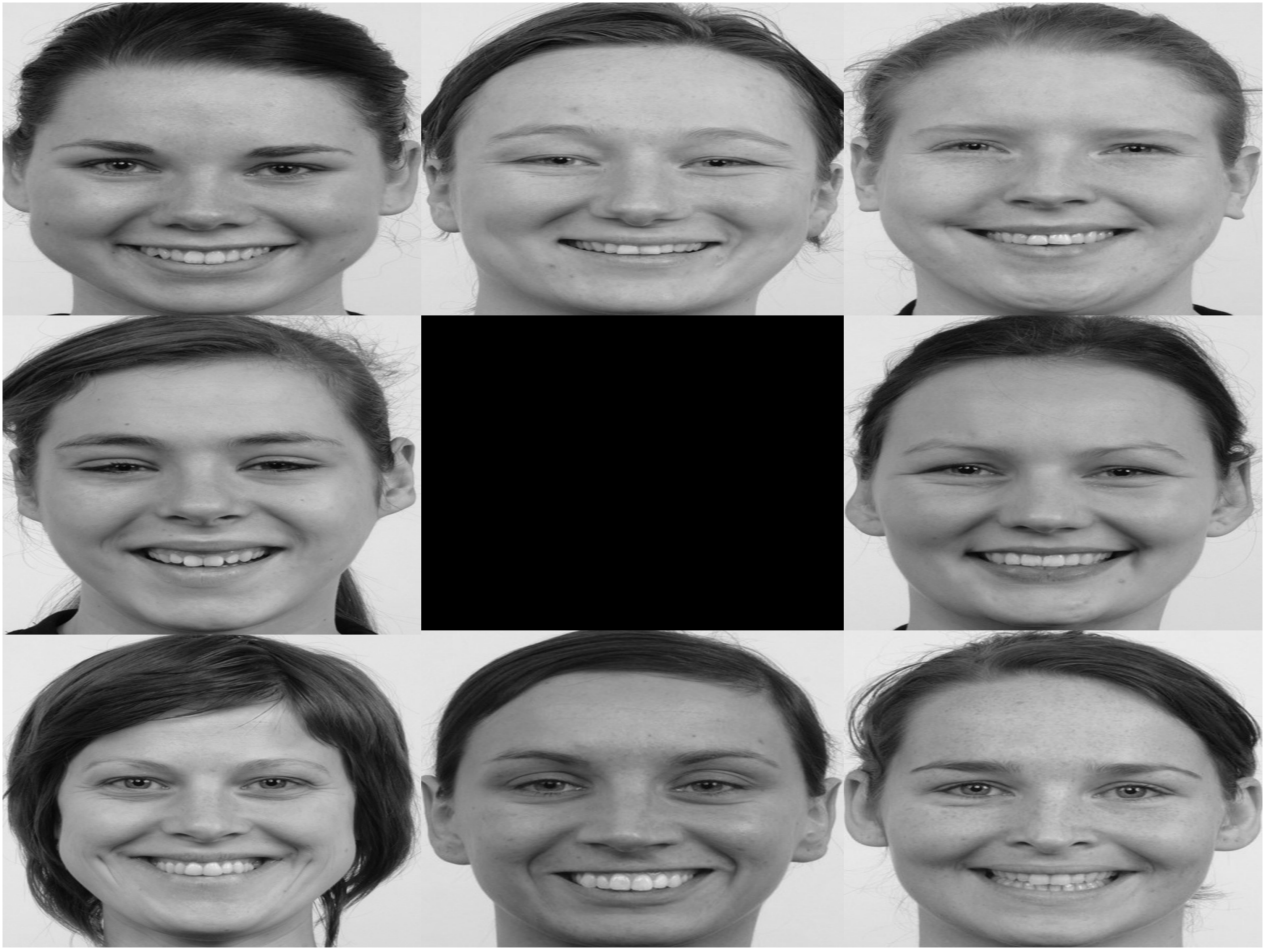
Example of a study slide task two (happy emotional expressions).

The visual stimuli consisted of series of slides each representing seven pictures of an unfamiliar women and one picture of the child’s mother. Task one consisted of neutral facial expressions (see figure 1; this is not the original image used in the study, but a similar image used for illustrative purposes only). A total of eight study slides were presented for ten seconds each, the location of the mother picture was randomized. Prior to the onset of each picture array, a white fixation cross was presented for 2 seconds in the middle of a black screen. The total duration of the task was 94 seconds. The second task exposed children to three different emotional expressions, i.e. happy, fearful, and angry faces. Each slide consisted of one emotional expression, exposing the child to one emotional expression a time (see figure 2; this is not the original image used in the study, but a similar image used for illustrative purposes only). The three emotional expressions were shown three times leading to nine study slides presented for ten seconds each, and each slide was preceded by a fixation cross for two seconds. Total duration of the task was 106 seconds. The location of mother’s picture was again randomized. To insure children were exposed to valid facial expressions in terms of the emotions mothers were supposed to convey, 33 children were asked after the eye-tracking tasks to circle the word that best matched the emotional expression of all pictures in task two. In addition to the six basic emotions, children could also choose ‘neutral’ and ‘indistinct’. Happy and angry faces were accurately recognized by respectively 88.78 and 70.16% of the children. Fearful faces were only accurately recognized by 28.3%; faces were mainly rated as surprised (46.2%). Due to difficulties in recognizing the fearful faces, only the data of happy or angry emotional faces (six slides) will be analyzed.

For both tasks, photographs of the mothers were identical in size (720×450 pixels) and were taken with a digital camera at the beginning of the study. All environment characteristics and basic visual properties (e.g. white background, illumination) were kept identical. The pictures focused on the mother’s face and avoided bright colors (e.g. earrings, teeth). The mother was asked to show a neutral, happy and angry face and was given examples and instructions. The unfamiliar women’s pictures were from the Radboud faces database [25]. Seven women were selected out of the database mainly based on the age criterion; to look old enough to represent one of the mothers. However, with each additional mother-child dyad being recruited, database-pictures were systematically replaced by pictures from other participants to increase stimulus similarity in terms of quality and background characteristics. So, from child two onwards the pictures were replaced by self-made pictures. Thus, no stimuli from the Radboud dataset were in the study slides after participant seven.

Eye movement data were collected using the TobiiT60 Eye Tracker (Tobii, Danderyd, Sweden), a table mounted binocular eye tracker with a sampling rate of 60 Hz. This system registered the accuracy of the eye movements up to 0.5° of a visual angle and stimuli were presented on a 17″ TFT monitor (1280×1024 pixels), connected to an IBM compatible laptop.

### Questionnaire measures

#### Attachment Security

Attachment security was measured with the continuous Trust and Communication subscales of the People In My Life Questionnaire [PIML; 26], which is designed to measure 10 to 12-year-old children’s representations of attachment figures. Unpublished data has shown that the questionnaire can be reliably used from 8-years onwards. Children responded on a 4-point Likert-scale ranging from 1 (almost never true) to 4 (almost always true). The PIML consists of three subscale, trust, communication, and alienation. Trust provides a measure of positive affective and cognitive experience associated with accessible and responsive attachment figures. The subscale consists of 10 items, e.g. I trust my mother. In contrast, alienation measures the negative affective and cognitive experiences associated with unresponsive or inconsistently responsive attachment figures and consists of five items (e.g., I feel angry with my mother). Third, five items (e.g. My mother listens to what I have to say) measure the subscale communication; the behavioral interactions between children and attachment figures [27]. Factor loadings suggested that attachment largely consists of trust in an individual as well as communication with that individual. Alienation was only moderately associated with attachment, suggesting that alienation is a measure that more strongly reflects the characteristics of the person rather than the experiences with that person in the relationship [26]. As reliability of the subscale alienation in different samples is not sufficient, ranging from .63 in a Flemish population (unpublished work), to .65 in an American sample [26], only the trust and communication subscales were summarized in the present study to measure secure attachment. Cronbach’s alpha of Attachment Security in the current sample was .81.

#### Attachment Avoidance and Attachment Anxiety

The Experiences in Close Relationships Scale-Revised (ECR-R) [28], was used to measure attachment anxiety and avoidance towards the mother. This child-friendly version of the ECR-R [28] has been developed by Brenning and colleagues [29] for children from 8 years onwards and has demonstrated good psychometric properties in a group of 8–14 years-olds. The anxiety scale (18 items) taps into feelings of fear of abandonment and strong desires for interpersonal merger (e.g., I worry about being abandoned). The avoidance scale (18 items) taps into discomfort with closeness, dependence, and intimate self-disclosure (e.g., I prefer not to show how I feel deep down). Items are rated on a 7-point scale ranging from ‘*not at all*’ to ‘*very much*’. Cronbach’s alpha in the current study was .78 and .82 for attachment anxiety and avoidance, respectively.

#### Control variables

The State-Trait Anxiety Inventory for Children [30], and the Children’s Depression Inventory [31] were administered. Satisfactory reliability was found for both trait anxiety (Cronbach’s alpha .84) and total depressive symptoms (Cronbach’s alpha .74).

### Procedure

Children were recruited out of schools in fourth, fifth and sixth grades of elementary school in Flanders, corresponding to ages ranging from 8–12, with flyers inviting them to come to the university to participate in research. Volunteering mothers and children were informed about the content of the study over phone and invited to the lab at the faculty. Response rate was approximately 10%. Parents and children were told that we were interested in how children process faces of their mother and unfamiliar women with different emotional expressions. They were informed that their pictures would be used in the computer assignment but would be erased afterwards. Arriving at the lab, mother and child were seated in two different rooms. All participating parents gave their informed consent for both taking photos and the test procedure for their child. After signing the informed consent, pictures from mother were taken. Next, mother completed a demographic form. The child was personally informed about the study and about their right to refuse participation. All children choose to participate. Then, children received individual instructions about the questionnaires. Children were told that the test leader was always available during the testing session to answer any question. Children started to fill out the questionnaires and if necessary, questionnaires were read out aloud.

Meanwhile photos were integrated in the study slides behind a little wall, withholding children to see the preparation of the study slides. Next, participants were seated at a distance of 60 cm in front of the computer screen with the chin placed comfortably in a chin rest to reduce movement. Calibration was repeated until the eye tracker was recording line of visual gaze within 1° of visual angle for each calibration point. Calibration was repeated until this criterion was met. Once calibration was successful, children were instructed via the computer screen to view the images naturally. All children were tested individually. The study was approved by the Institutional Review Board of the Faculty of Psychology and Pedagogical Sciences at Ghent University. At the end of the experimental session participants were debriefed and received two cinema-tickets for their participation.

### Eye movement processing and analysis

Location, time and duration of all fixations were analyzed. Eight different areas of interest (AOI) were defined for each study slide. Each AOI represented one of the eight pictures in the matrix, with some space between the AOI’s.

Following dependent variables were calculated for both the mother and the unfamiliar women:

#### Total viewing time

This is the total duration a participant fixated on an AOI independent of attentional shifts. A gaze fixation should be 60 ms to be classified as a fixation. The total viewing time was calculated by averaging the total viewing time over AOIs and over the study slides.

#### Maintained attention

This is the extent of time a participant’s gaze remains fixated within the boundaries of a particular AOI, taking into account the amount of attentional shifts, indicating maintenance of attention. This was calculated by dividing the total viewing time on an AOI by the amount of fixations within this AOI. This proportion was averaged over AOIs (one for mother, seven for unfamiliar women) and over the study slides.

#### Relative visit frequency

This is the proportion of visual visits of a participant on a particular AOI relative to the total visits over all AOIs. We averaged this proportion over AOIs (one for mother, seven for unfamiliar women) and over the study slides to calculate the relative visit frequency on mother and on the unfamiliar women.

### Data Analyses

Of all participants, one child (1.6%) did not fill out the attachment security questionnaire, no other data were missing. Little’s [32] MCAR-test produced a normed χ^2^ (χ^2^/df) of 1.66. According to Bollen [33], this indicates that the data were likely missing at random, and as a consequence, missing values could be estimated. To do so, we used the Estimation Maximization. The in- or exclusion of the subject with the missing data point does not change the results.

Performances on the first attentional task were examined by a within-subjects analysis of covariance (ANCOVA) with Familiarity (mother vs. unfamiliar) as within-subjects variable and the eye-movement variables as dependent variables. To investigate the specific moderating role of an attachment style, the three attachment measures (Attachment Security, Attachment Avoidance, and Attachment Anxiety) were added separately as covariates of interest to the ANCOVA. In all analyses age was added as covariate and gender as between-subject-variable. With regard to the second emotional task, a 2 (Familiarity: mother vs. unfamiliar) × 2 (Emotion: happy and angry) within-subjects ANCOVA was conducted on the eye-movement variables. Like in task one, age was added as covariate and gender as between-subject-variable. Again, as for the first task, the three attachment measures served as covariates of interest. For each dependent variable, we will first report the main effects, followed by the interaction-effects with the variables of interest, which examine our research questions. Finally, we will report on gender and age-effects. Effects are considered significant if the *p*-value is below .05, and trending if p-value is below .065.

Finally, to interpret the ANCOVA’s difference scores (e.g. eye movement data mother minus eye movement data unfamiliar women) were calculated; the higher the difference score (DS), the more the child was looking at mother in comparison with the unfamiliar women. Correlational analyses were conducted between the attachment measures and the difference scores enabling us to interpret significant interactions between attachment and Familiarity.

To examine the extent to which trait anxiety and depressive symptoms may have influenced the findings, analyses were rerun with trait anxiety and depressive symptoms as covariates.

## Results

### Preliminary analyses

Saccades are commonly non-normally distributed [34]. Therefore, the dependent variables were logarithmic transformed before subjecting to ANCOVA. Outliers for each task and each variable were deleted out of the analyses (+/−2 SD). Moreover, we checked for bivariate outliers. No influential cases could be detected in the analyses (all cook’s distances <.28). See table 1 and 2 for means and standard deviations and correlational analysis for respectively task one and 2 (based on non-transformed data). Effects of age and gender on the saccades were analyzed using a Multivariate analysis with saccades as dependent variables, gender as fix-factor and age as covariate. For task one, there was a significant effect of gender on *total viewing time* to unfamiliar faces, *F*(1, 52) = 5.70, *p*<0.05, *η_p_* ^2^ = .10. Boys viewed significant less at unfamiliar faces (*M* = .72, *SD* = .22) than girls (*M* = .84, *SD* = .14). For task two, age had a significant effect on the dependent variables of task two, *F*(8, 38) = 2.74, *p*<0.05, *η_p_* ^2^ = .37. More specifically, age had a significant effect on *maintained attention* of the unfamiliar women with happy, *F*(1, 45) = 4.06, *p* = .05, *η_p_* ^2^ = .08, and angry, *F*(1, 45) = 4.06, *p*<.01, *η_p_* ^2^ = .18, facial expressions. The older the child, the less maintained attention at the unfamiliar’s happy, *r*(52)* = *–.23, *ns,* and angry, *r*(52)* = *–.37, *p*<.01, faces.

**Table 1:** Means and Standard Deviations of total viewing time, maintained attention, and relative visit frequency for mother’s and unfamiliar’s faces and correlational analyses between the variables for task one.

	1	2	3	4	5	6
1. Total viewing time Mother	1					
2. Total viewing time Unfamiliar	–.50**	1				
3. Maintained Attention Mother	.64**	–.55**	1			
4. Maintained Attention Unfamiliar	–.05	.12	.25	1		
5. Visit Frequency Mother	.64**	–.08	.44**	–.15	1	
6. Visit Frequency Unfamiliar	–.64**	.08	–.44**	.15	–1	1
Mean (*SD*)	1.90 (1.06)	.79 (.18)	.37 (.14)	.28 (.04)	82.94 (2.82)	17.06 (2.82)

*p<.05*;*

**p<.01.

**Table 2:** Means and Standard Deviations of total viewing time, maintained attention, and relative visit frequency for mother’s and unfamiliar’s faces and correlational analyses between the variables for task two.

	1	2	3	4	5	6	7	8	9	10	11	12
1. TVTMH	1											
2. TVTMA	.45**	1										
3. TVTUH	–.48**	–.01	1									
4. TVTUA	.08	–.30*	.37**	1								
5. MHMA	.66**	.36*	–.32*	.07	1							
6. MAMA	.40**	.62**	.03	–.09	.55**	1						
7. MAUH	.09	–.06	.15	.06	.25	.17	1					
8. MAUA	.13	–.23	–.04	.25	.32*	.05	.42**	1				
9. VFMH	.50**	.14	–.26	–.11	.29*	.34*	.06	.09	1			
10. VFMA	.22	.54**	–.12	–.29	.20	.20	–.20	–.31*	.29*	1		
11. VFUH	–.50**	–.14	.26	.11	–.29	–.34	–.06	–.09	–1.00**	–.29*	1	
12. VFUA	–.22	–.54**	.12	.29	–.20	–.20	.20	.31	–.29*	–1.00	.29*	1
Mean (*SD*)	1.51 (.89)	1.51 (.72)	.88 (.14)	.87 (.14)	.32 (.10)	.32 (.08)	.29 (.05)	.28 (.04)	83.16 (.03)	83.22 (.03)	16.84 (.03)	16.78 (.04)

TVTMH: Total Viewing Time Mother Happy, TVTMA: Total Viewing Time Mother Angry, TVTUH: Total Viewing Time Unfamiliar Happy, TVTUA: Total Viewing Time Unfamiliar Angry, MAMH: Maintained Attention Mother Happy, MAMA: Maintained Attention Mother Angry, MAUH: Maintained Attention Unfamiliar Angry, MAUA: Maintained Attention Unfamiliar Angry, VFMH: Visit Frequency Mother Happy, VFMA: Visit Frequency Mother Angry; VFUH: Visit Frequency Unfamiliar Happy; VFUA: Visit Frequency Unfamiliar Angry.

*p<.05*;*

**p<.01.

With regard to the attachment variables, outliers (+/−2 *SD*) were deleted (*N* = 2). Attachment Security had a mean of 53.97 (*SD* = 4.38) and correlated significantly with Attachment Avoidance, *r*(60) = –.70, *p*<.001, and Attachment Anxiety, *r*(60) = –.28, *p*<.05. Attachment Avoidance (*M = 1.94, SD = .62*) correlated significantly with Attachment Anxiety (*M = 2.44, SD = .73*), *r*(60)* = *.38, *p*<.01. Effects of age and gender on the attachment variables were analyzed using a Multivariate analysis with attachment types as dependent variables, gender as fix-factor, and age as covariate. Gender was significantly related to Attachment Security, *F*(1, 59) = 4.73, *p*<.04, *η_p_* ^2^ = .07. Girls (*M* = 54.06, *SD* = 3.54) had significantly higher scores on Attachment Security than boys (*M* = 51.33, *SD* = 5.04).

For task one, in the ANCOVA’s with Familiarity (mother vs. unfamiliar) as within-subjects variable, age as covariate, and gender as between-subject-variable, there was no main effect of Familiarity for *total viewing time*, *F*(1,52) = 3.37, *ns*, *η_p_^2^* = .06, and for *maintained attention*, *F*(1,55) = .15, *ns*, *η_p_^2^* = .000. For *relative visit frequency*, a significant main effect of Familiarity was found, *F*(1, 54) = 19.44, *p*<.001, *η_p_* ^2^ = .27, indicating more visits on mother (*M* = 82.94, *SD* = 2.82) in comparison with the unfamiliar women (*M* = 17.06, *SD* = 2.82). No effects were found for age, and gender (all *F*s<2.85, *η_p_^2^*<.05). For task two, the ANCOVA’s, with Familiarity (mother vs. unfamiliar) and Emotion (happy vs. angry) as within-subjects variables, age as covariate, and gender as between-subject-variable, showed no main effect of Familiarity for *total viewing time, F*(1,49) = .86, *ns*, *η_p_* ^2^ = .017, and *maintained attention*, *F*(1,51) = .48, *ns*, *η_p_* ^2^ = .009. No main effects for Emotion on *total viewing time*, *F*(1,49) = .003, *ns*, *η_p_* ^2^ = .000, and *maintained attention*, *F*(1,51) = 1.37, *ns*, *η_p_* ^2^ = .03, were found. Further, there were no significant interaction effects (all *F*s<3.55, *η_p_* ^2^s<.065). Analysis of visit frequency was not possible in task two because viewing data on two emotions are complementary and add up to 100.

### Attachment and eye movements towards neutral faces of mother and unfamiliar women (Task one)

**Figure 3:**
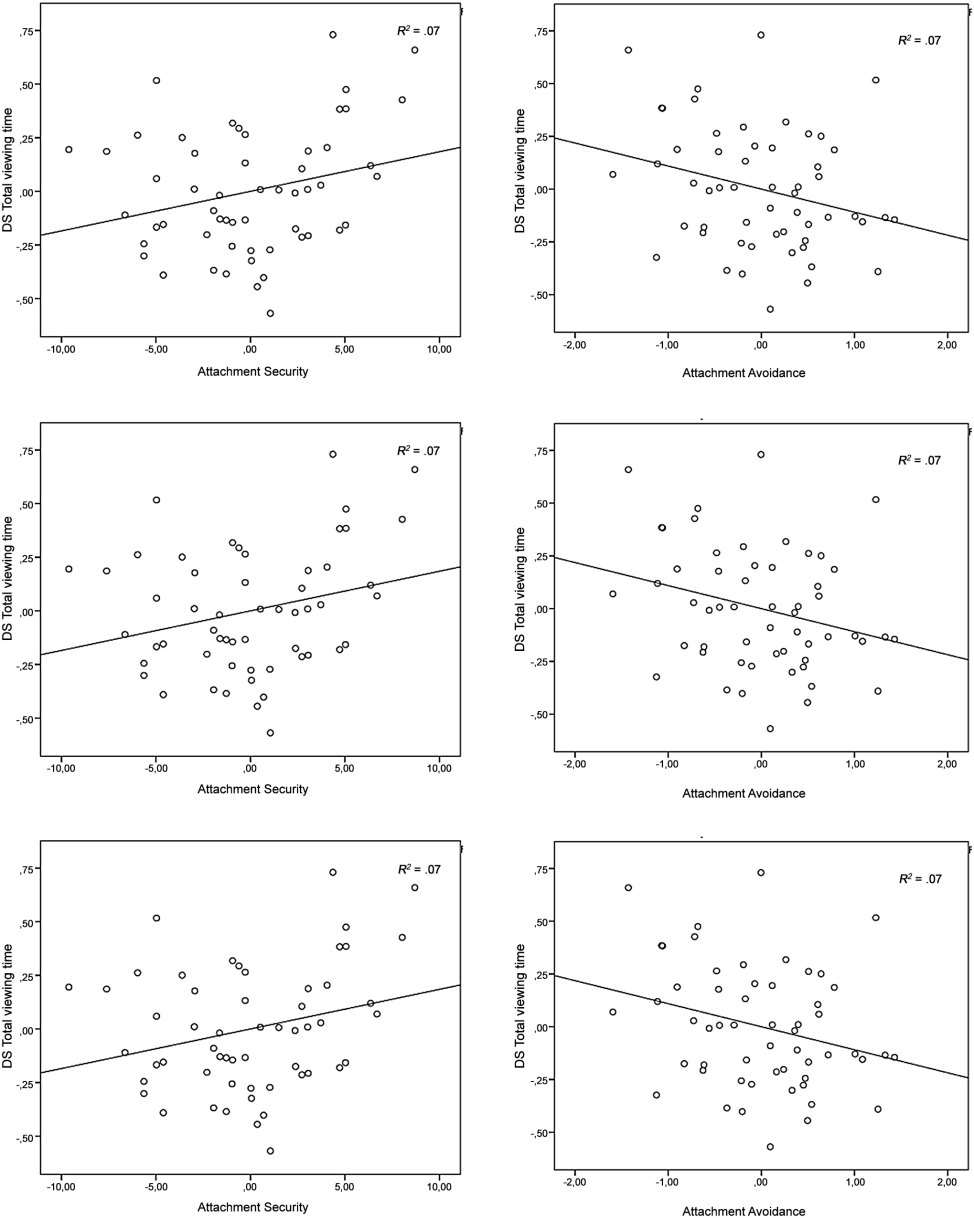
DS *total viewing time* (task one) plotted against Attachment Avoidance and Attachment Security, controlled for age and gender. Solid line represents the regression line.

For the first dependent variable *total viewing time,* a main effect of Familiarity was found in the ANCOVA with the covariate Attachment Avoidance, *F* (1, 51) = 5.97, *p*<.05, *η*_p_^2^ = .11, indicating a longer *total viewing time* on mother (*M* = 1.90, *SD* = 1.06) in comparison with the unfamiliar women (*M* = .79, *SD* = .18). Main effects in the models with the covariates Attachment Anxiety, *F* (1, 51) = 3.23, *ns*, *η*_p_^2^ = .06, and Attachment Security, *F* (1, 51) = .053, *ns*, *η*_p_^2^ = .001, were not significant. Second, Familiarity significantly interacted with Attachment Avoidance, *F*(1,51) = 4.09, *p*<.05, *η_p_^2^* = .07, and trend significantly with Attachment Security, *F*(1, 51) = 3.81, *p*<.06, *η_p_^2^* = .04. The more avoidantly attached, the less the child fixated on mother as compared to the unfamiliar women (*r*(51) = –.27, *p*<.05). The more securely attached, the more the child fixated on the mother than on the unfamiliar women (*r*(51) = .26, *p*<.06). Scatterplot data are provided for visual inspection (Figure 3). No interaction with Attachment Anxiety, *F*(1, 51) = .08, *ns*, *η_p_^2^* = .001, was found. Moreover, an interaction effect was found between Familiarity and Gender in the models with the covariates Attachment Security, *F*(1,51) = 5.23, *p*<.05, *η_p_^2^* = .09, and Attachment Avoidance, *F*(1,51) = 4.25, *p*<.05, *η_p_^2^* = .08. Boys (*M* = 2.09, *SD* = 1.29) looked at mother’s face more than girls (*M* = 1.75, *SD* = .85). In addition, boys (*M* = .72, *SD* = .22) looked at unfamiliar’s faces less than girls (*M* = .84, *SD* = .14). No other effects of age and gender were found (all *F*’s<2.80, *η_p_^2^*<.05).

**Figure 4:**
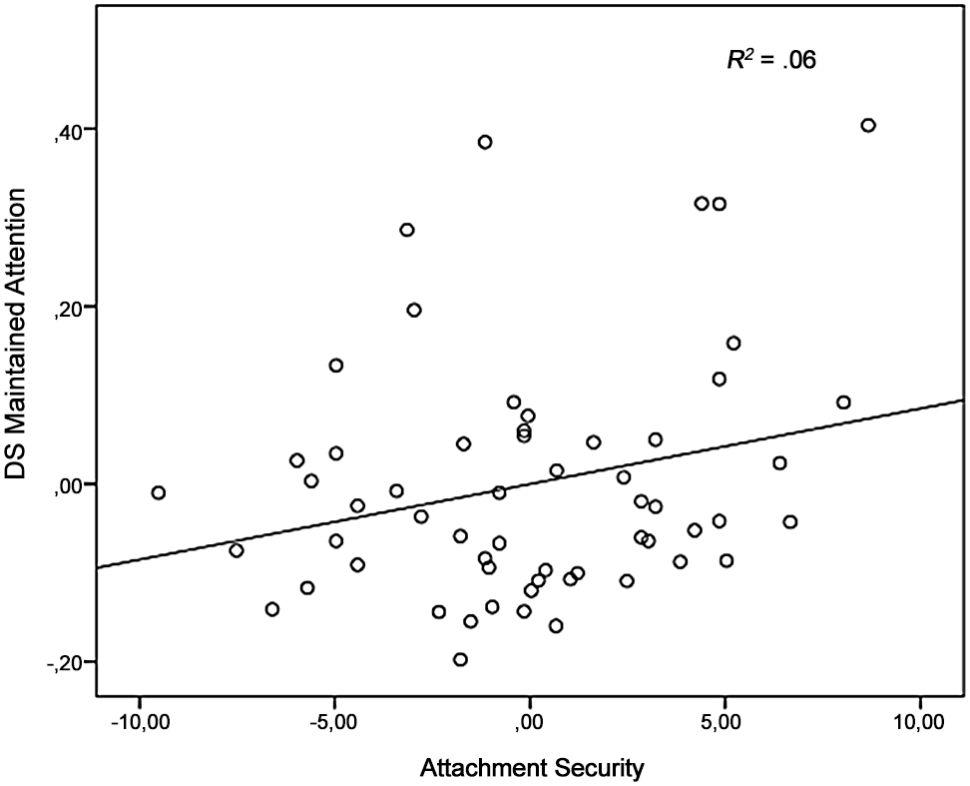
DS *maintained attention* (task one) plotted against Secure Attachment, controlled for age and gender. Solid line represents the regression line.

For the dependent variable *maintained attention,* no main effects of Familiarity were found in the models with the attachment variables (all *F*’s<2.13, *η_p_^2^*<.04). Familiarity interacted trend significant with Attachment Security, *F*(1,54) = 3.68, *p* = .06, *η_p_^2^* = .06. Follow-up correlational analyses showed that the more securely attached the child, the more maintained attention on the mother in comparison with the unfamiliar women (*r*(54) = .25, *p* = .06). Scatterplot data are provided for visual inspection (Figure 4). No interaction between Familiarity and Attachment Avoidance, *F*(1,54) = 3.02, *ns*, *η_p_^2^* = .05, and Attachment Anxiety, (*F* (1, 54) = .03, *ns*, *η_p_^2^* = .001, were found. No effects of age and gender were found in the ANCOVA’s (all *F*’s<3.05, *η_p_^2^*<.05).

**Figure 5:**
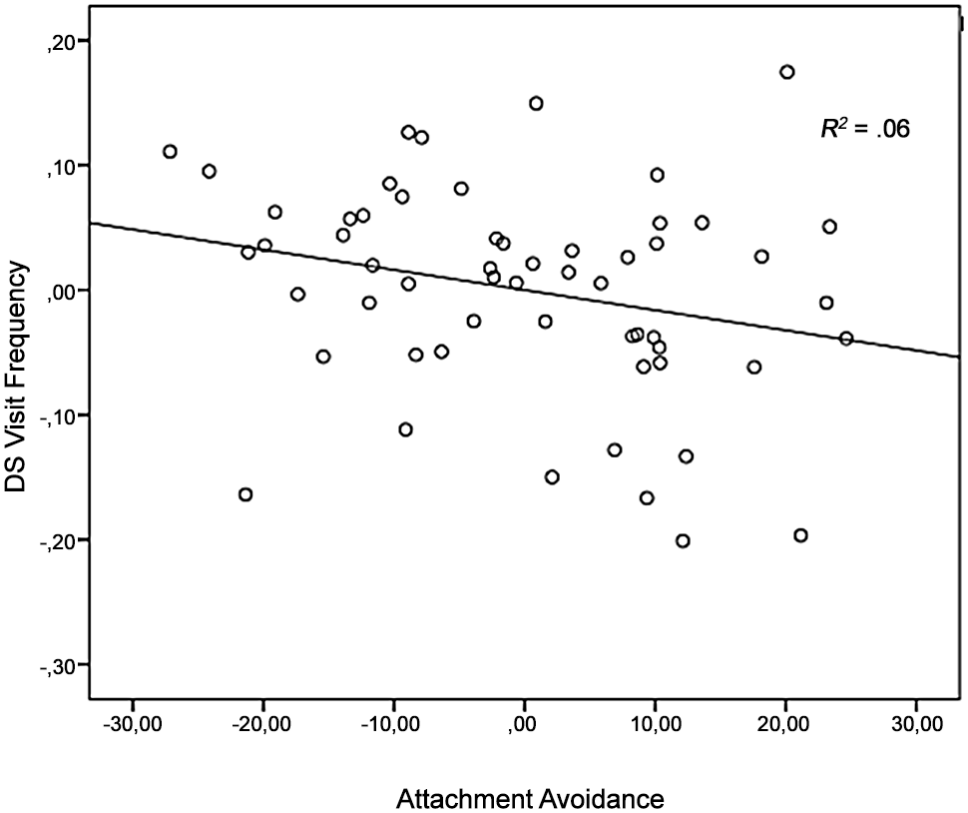
DS *visit frequency* (task two) plotted against Attachment Avoidance, controlled for age and gender. Solid line represents the regression line.

For *relative visit frequency*, a main effect was found in the ANCOVA with Attachment Security, *F* (1, 53) = 22.93, *p*<.001, *η_p_^2^* = .31, Attachment Avoidance, *F* (1, 53) = 22.93, *p*<.001, *η_p_^2^* = .31, and Attachment Anxiety, *F* (1, 53) = 20.19, *p*<.001, *η_p_^2^* = .28, indicating more visits on mother (*M* = 82.94, *SD* = 2.82) in comparison with the unfamiliar women (*M* = 17.06, *SD* = 2.82). Furthermore, Familiarity interacted trend significant with Attachment Avoidance, *F*(1,53) = 3.65, p<.065, *η_p_^2^* = .06. The more avoidantly attached, the less visits on mother relatively to visits on the unfamiliar (*r*(53)* = *–.25). Scatterplot data are provided for visual inspection (Figure 5). There were no significant interactions with Attachment Security, *F*(1,53) = 3.18, *ns*, *η_p_^2^* = .06, and Attachment Anxiety, *F*(1,53) = .61, *ns*, *η_p_^2^* = .01. No effects of age and gender were found (all F’s<.09, *η_p_^2^*<.002).

### Attachment and eye movements towards emotional faces (task two)

**Figure 6:**
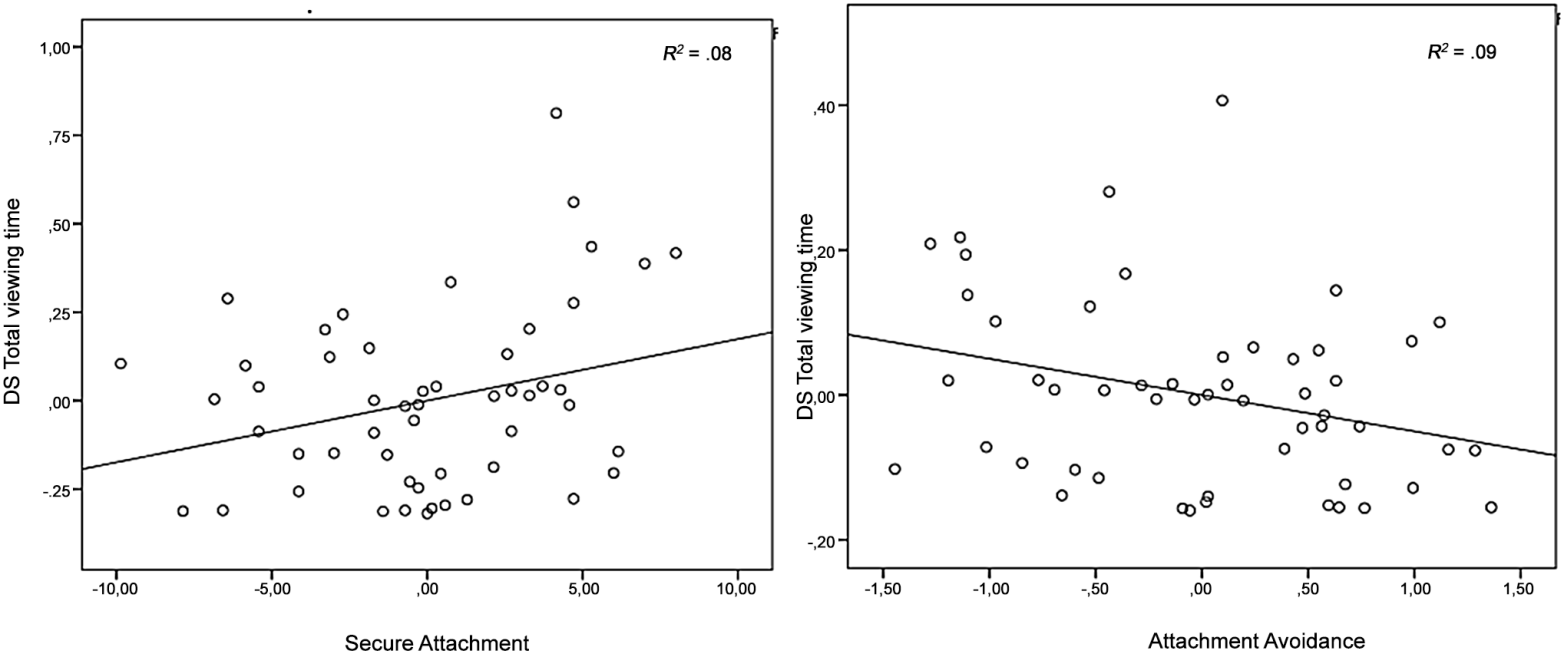
DS *total viewing time* (task two) plotted against Attachment Security and Attachment Avoidance, controlled for age and gender. Solid line represents the regression line.

For the dependent variable *total viewing time*, analyses resulted only in a significant main effect of Familiarity adding Attachment Security, *F*(1,48) = 4. 47, *p*<.05, *η_p_^2^* = .09, indicating that over all emotional expressions children had a longer total viewing time on mother (*M* = 3.03, *SD* = 1.38) than on the unfamiliar women (*M* = 1.75, *SD* = .24). There were no main effects in the models with Attachment Anxiety, *F*(1,48) = .68, *ns*, *η_p_^2^* = .01, and Attachment Avoidance, *F*(1,48) = 4.54, *ns*, *η_p_^2^* = .05. Moreover, there were no main effects of Emotion (all F’s<3.24, *η_p_^2^*<.06), nor were interactions found between Familiarity and Emotion (*F*(1,48) = 4.54, *ns*, *η_p_^2^* = .05). Further, for *total viewing time*, the interactions between Attachment Security and Familiarity, *F*(1,48) = 4.31, *p*<.05, *η_p_^2^* = .08, and between Attachment Avoidance and Familiarity, *F*(1,48) = 4.54, *p*<.05, *η_p_^2^* = .09, were significant. The more securely attached, the longer the child looking at mother than at the unfamiliar women (*r*(48) = .29, *p*<.05), regardless of the emotional expression (happy and angry). The more avoidantly attached, the more reduced *total viewing time* on mother than on the unfamiliar women (*r*(48) = –.29, *p*<.05). Scatterplot data are provided for visual inspection (figure 6). No interaction-effect between Familiarity and Attachment Anxiety was found, *F*(1,48) = .00, *ns*, *η_p_^2^* = .003. Moreover, no significant interaction effects were found between Emotion and the attachment variables (all F’s<1.71, *η_p_^2^*<.03), nor was the three-way-interaction between Familiarity, Emotion, and Attachment significant (all F’s<2.06, *η_p_^2^*<.04). Further, no significant effects of age and gender were found (all F’s<2.27, *η_p_^2^*<.05).

**Figure 7:**
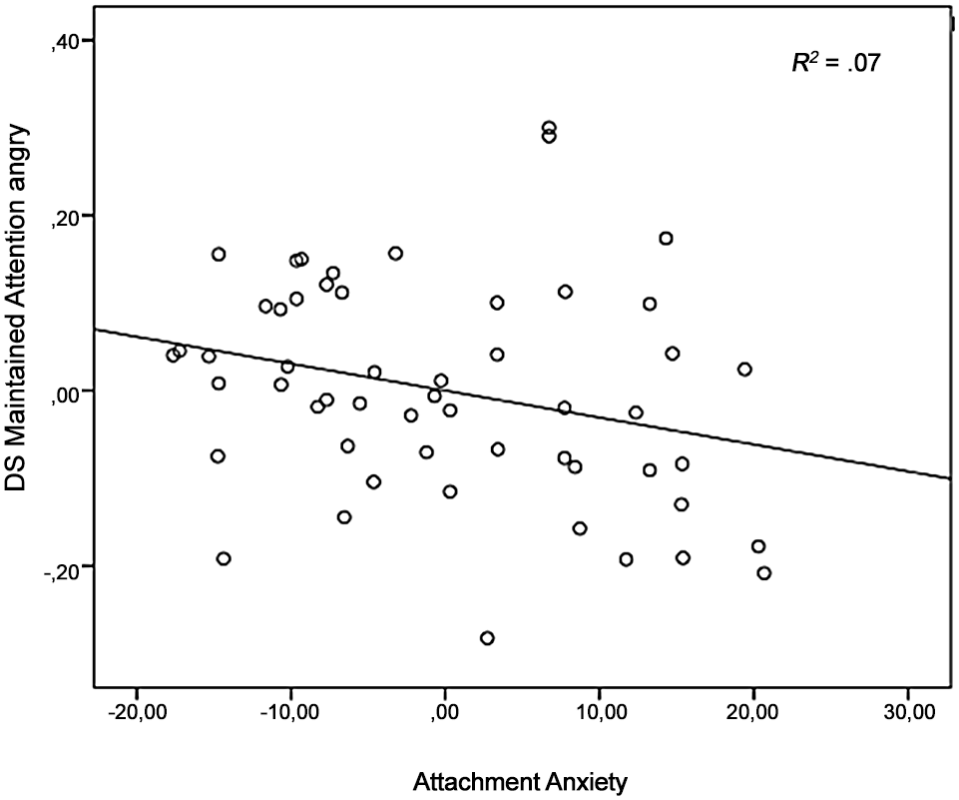
DS *maintained attention* angry faces (task two) plotted against Attachment Anxiety, controlled for age and gender. Solid line represents the regression line.

For the dependent variable *maintained attention*, no main effects of Familiarity or Emotion, nor an interaction effect between Familiarity and Emotion were found in the models (all F’s<2.79, *η_p_^2^*<.05). Further, no significant interaction effects between Familiarity and Attachment Security, *F*(1, 50) = 2.68, *ns*, *η_p_^2^* = .05, nor with Attachment Avoidance, F(1, 50) = .62, *ns*, *η_p_^2^* = .01, and Attachment Anxiety, (F(1, 50) = .54, *ns*, *η_p_^2^* = .01, were found. Furthermore, there were no significant interaction effects between Emotion and the attachment variables (all F’s<.62, *η_p_^2^*<.01). Adding Attachment Anxiety in the ANCOVA resulted in a significant three-way-interaction effect between Familiarity, Emotion and Attachment Anxiety, *F*(1, 50) = 4.59, p<.05, *η_p_^2^* = .10. Follow-up analysis indicated that there was a significant interaction between Familiarity and Attachment Anxiety on the angry faces, *F*(1,50) = 3.95, *p* = 0.05, *η_p_^2^* = .07. The more anxiously attached, the less maintained attention on mother her angry face (*r*(50) = –.27, *p* = .05). Scatterplot data are provided for visual inspection (figure 7). Three-way-interaction effects with Attachment Avoidance, *F*(1, 50) = .48, *ns*, *η_p_^2^* = .01, and Attachment Security, *F*(1, 50) = .13, *ns*, *η_p_^2^* = .003, were not significant. No effects of age and gender were found (all F’s<3.12, *η_p_^2^*<.06).

### Additional analyses

When trait anxiety and depressive symptoms were taken into consideration in task one, for *total viewing time*, the interaction between Familiarity×Attachment Security remained trend significant, respectively F(1,44) = 3.76, *p*<.06, *η_p_^2^* = .07. However, Familiarity×Attachment Avoidance turned into trend significance, *F*(1,44) = 3.43, *p*<.07, *η_p_^2^* = .07. For *Maintained Attention*, the interaction between Familiarity×Attachment Security remained trending, *F*(1,53) = 3.72, *p*<.06, *η_p_^2^* = .07. For *Visit Frequency,* the interaction between Familiarity×Attachment Avoidance turned into non-significance, *F*(1,49) = 2.23, *ns*, *η_p_^2^* = .04. For task two, when trait anxiety and depressive symptoms were taken into consideration, for *total viewing time*, the interaction between Familiarity and Attachment Security, *F*(1,44) = 4.53, *p*<.055, *η_p_^2^* = .08, and Attachment Avoidance, *F*(1,44) = 4.49, *ns*, *η_p_^2^* = .09, remained significant. For *maintained attention*, the three-way-interaction between Attachment Avoidance×Familiarity×Emotional expression remained significant when adding trait anxiety and depressive symptoms, *F*(1,45) = 4.69, *p*<.05, *η_p_^2^* = .09. These data suggest that variation in trait anxiety and depressive symptoms only slightly impacted the direction of the findings.

## Discussion

The present study investigated attentional biases in the processing of faces of mother and unfamiliar women by monitoring the eye movements of children using a naturalistic viewing task. We focused on how attachment affects the attentional processing of attachment-related neutral (task one) and emotional (task two) information.

Confirming the defensive exclusion hypothesis [1]–[6] and in line with previous research studying attention to social information using reaction-time tasks in adults [16] and using eye movements in infants and toddlers [22], findings suggest an open manner of processing all attachment-related information in securely attached children, and a defensive exclusion of this information in more avoidantly attached children. Task one revealed that secure attachment was related to a longer total viewing time and more maintained attention on mother’s neutral face. Attachment avoidance was characterized by a reduced total viewing time on mother’s neutral face. Moreover, more avoidantly attached children tended to make less visits on mother’s face relatively to the visits on unfamiliar’s face. In task two, regardless of the emotional valence, the total viewing time on the mother’s face was significantly longer for more securely attached children. Attachment avoidance, in contrast, was associated with a reduced total viewing time on the mother’s face regardless of emotional valence. Results replicated and extended findings that more avoidantly attached children excluded both negative, and positive attachment-relevant information, while more securely attached children on the other hand paid more attention to attachment-related positive and negative information.

At first sight, these results seem in contrast with the findings of Bosmans et al. [8], which indicated that low securely attached children showed more maintained attention and engagement towards mother’s faces in comparison with unfamiliar women’s faces. However, these different findings could reflect differences in the employed paradigms. Bosmans and colleagues [8] used an exogenous cueing task. Children were instructed to react as fast and as correct as possible on valid and invalid cued targets preceded by a picture of either mother or an unfamiliar woman. In contrast, children freely explored the visual stimuli presented in our study. Due to these task-related differences, both tasks were measuring different aspects of attentional processing. More specifically, the exogenous cueing task measured engagement and disengagement, while in the current study spontaneous looking behavior was measured. Thus, more securely attached children appear to be able to disengage from their mother’s face when they are instructed to. However, when more securely attached children can freely view the environment, they appear to prefer paying attention to their mother rather than to unfamiliar women. Furthermore, highly securely attached children could easily disengage from both their mother’s and the unfamiliar women’s faces [8], which could indicate that the open information processing style of securely attached children is not only characteristic for attachment-related information, but is also applicable to non-attachment related stimuli. Consequently, we hypothesize that more securely attached children are characterized by an open attentional processing style, leading to more opportunities to explore the world [1].

Furthermore, results seem to indicate a difference in the attentional processing of mother depending on the insecure attachment style. Contrary to the theoretical prediction that all insecurely attached children exclude attachment-related information [1], little evidence was found for a biased attentional processing effect in attachment anxiety. Only one link was significant: attachment anxiety was related to reduced maintained attention on mother’s angry face in comparison with the unfamiliar women’s angry faces. This lack of clear links with attachment anxiety is in line with other research [22] and could result from the hyperactivating strategies related with attachment anxiety [35]. It is less obvious that these characteristics might serve to shut out attachment-related information. However, neither do these characteristics lead to a vigilance effect [7]. Given that attachment anxiety originates from a history of unsupportive attachment experiences, it is more likely that one will first defensively exclude information that potentially will lead to fear. Following Mogg and Bradley [36], we assume that longer stimulus presentations evoke ruminative responses leading to a shift towards threat, followed by directing attention away from the threat. This shifting of attention toward and away from the threat might be reflected in a significant negative relation with maintained attention towards the mother. To fully disentangle the biased processing of attachment-related information, further research should take into account the time-course of attention [37].

Studying attachment-related information processing in children might bear implications for propensity for psychopathology. Excluding attachment information can be functional in the short-term for insecurely attached children, as it avoid painful feelings related with the attachment figure and can help to seek for alternative sources of security. However, in the long term, potentially corrective experiences regarding care and comfort are excluded as well, leading to the maintenance of insecurity in current and future relations [35]. So far, interventions to improve the attachment bond between mother and child mainly target the sensitivity and responsiveness of the parent [38]. However, if the child defensively excludes the caregiver, more sensitive and responsive parenting behavior might not be noticed and consequently insecure attachment representations could remain unaltered in spite of parents’ efforts. It is useful to evaluate if novel therapies based on visual selective attention patterns, like Attentional Bias Modification Treatment (ABMT), could be developed to improve attachment quality.

Some limitations of the current study should be noted. First, the use of self-reported measurements likely led to an underestimation of attachment insecurity [39]. Although both ECR-RC [29] and PIML [26] have excellent psychometric properties, attachment researchers traditionally advocate the use of interview measures like the Child Attachment Interview [40]. However, recent research [41] has shown that self-reported attachment and narrative attachment measures are significantly correlated in childhood. These findings suggest that self-reported measures might be a valid way to measure attachment in this specific age-group. Notwithstanding this underestimation of attachment insecurity, it is encouraging that significant results were found suggesting that the use of interviews or more implicit measures could lead to larger effect sizes. Second, a smaller area of attachment-related information relative to unfamiliar information was represented on the slides. If only two faces were shown, effects could have been more prominent. However, showing seven unfamiliar women minimalized the risk of noise by similarities with mother, attractiveness or salient facial characteristics. Third, the present study was conducted in a normal functioning population, limiting generalization to clinical groups. In addition, it could be that a more securely attached group was targeted in the study. This might have led to a restriction of range, meaning that effects could be more prominent in a more diverse group.

The current study contributes to the existing literature for at least four reasons. First, the current study was the first study that tracks eye movements using an accurate eye tracking monitor to address the influence of attachment-related expectations on the processing of attachment-related neutral and emotional information [6]. Furthermore, a validated and more ecological task paradigm [17] was used, including pictures with an idiosyncratic meaning. Third, we targeted an understudied age group. Generalizing results from another age group is hard since there is evidence that biases vary depending on cognitive- and perceptual maturity [42]. Last, by checking for both trait anxiety, and depressive symptoms, we could exclude that associations were driven by current mood state rather than by attachment differences.

In general, the current study confirmed that differences in attachment style affect the attentional processing of attachment-related information in late childhood and provided additional evidence for the defensive exclusion hypothesis [1]. Attachment Security was characterized by openly processing all attachment-related information, even when potentially painful. More avoidantly attached children, in contrast, had a biased manner of processing all attachment-relevant information. Their attention to the attachment figure’s neutral, positive and negative information was limited. Finally, more anxiously attached children could not be systematically characterized by a biased manner of processing attachment-relevant information.
